# Gene expression dataset for whole cochlea of *Macaca fascicularis*

**DOI:** 10.1038/s41598-018-33985-9

**Published:** 2018-10-22

**Authors:** Hideki Mutai, Fuyuki Miya, Hiroaki Shibata, Yasuhiro Yasutomi, Tatsuhiko Tsunoda, Tatsuo Matsunaga

**Affiliations:** 1 Division Hearing and Balance Research, National Institute of Sensory Organs, National Hospital Organization Tokyo Medical Center, 2-5-1 Higashigaoka, Meguro-ku, Tokyo 152-8902 Japan; 2 Department of Medical Science Mathematics, Medical Research Institute, Tokyo Medical and Dental University, 1-5-45 Yushima, Bunkyo-ku, Tokyo 113-8510 Japan; 3 Laboratory for Medical Science Mathematics, RIKEN Center for Integrative Medical Sciences, 1-7-22 Suehirocho, Tsurumi-ku, Yokohama, Kanagawa 230-0045 Japan; 4 Tsukuba Primate Research Center, National Institutes of Biomedical Innovation, Health and Nutrition, 1-1 Hachimandai, Tsukuba-shi, Ibaraki 305-0843 Japan; 5 Center for Development of Advanced Medical Technology, Jichi Medical University, 3311-1, Yakushiji, Shimotsuke-shi, Tochigi 329-0498 Japan; 6 Medical Genetics Center, National Hospital Organization Tokyo Medical Center, 2-5-1 Higashigaoka, Meguro-ku, Tokyo 152-8902 Japan

## Abstract

*Macaca fascicularis* is a highly advantageous model in which to study human cochlea with regard to both evolutionary proximity and physiological similarity of the auditory system. To better understand the properties of primate cochlear function, we analyzed the genes predominantly expressed in *M. fascicularis* cochlea. We compared the cochlear transcripts obtained from an adult male *M. fascicularis* by macaque and human GeneChip microarrays with those in multiple macaque and human tissues or cells and identified 344 genes with expression levels more than 2-fold greater than in the other tissues. These “cochlear signature genes” included 35 genes responsible for syndromic or nonsyndromic hereditary hearing loss. Gene set enrichment analysis revealed groups of genes categorized as “ear development” and “ear morphogenesis” in the top 20 gene ontology categories in the macaque and human arrays, respectively. This dataset will facilitate both the study of genes that contribute to primate cochlear function and provide insight to discover novel genes associated with hereditary hearing loss that have yet to be established using animal models.

## Introduction

Although the basic histological components of cochlear tissues are consistent among mammalian species^1^, each species has a unique range of auditory frequencies^2^ to perceive environmental change and communicate. This physiological variation can be explained not only by the morphological properties of conductive auditory organs such as the auditory canal, eardrum, and ear ossicles but also by the magnitude of expression of cochlea-specific genes. *Macaca fascicularis* (also called long-tailed, cynomolgus, or crab-eating macaque) is one of the best-studied nonhuman primate models for biomedical research; the entire genome has been sequenced, and most of the genes have been annotated^3,4^. The extremely high similarity between human transcripts and those of *M. fascicularis* as well as *Macaca mulatta* (rhesus macaque) has enabled investigators to study the gene expression profiles of macaque tissues using both the macaque and human microarray platforms^5^. The hearing range of *M. fascicularis* is from <0.1 to >32 kHz^6^, which overlaps with human hearing rage from <0.1 to 20 kHz. Therefore, *M. fascicularis* is considered a highly advantageous model to study human cochlea with regard to both the evolutionary proximity and physiological similarity of the auditory system.

To date, more than 100 genes have been identified that are associated with nonsyndromic hearing loss in humans^7^, and the number is increasing. While most of the genetic studies of cochlea have been carried out using rodents or other vertebrate species, the number of genetic studies using human cochlea is limited, mainly due to the difficulties in obtaining fresh cochlear tissues. Gene expression analysis from postmortem, formalin-fixed human cochlea is challenging due to fragmentation of the nucleic acids during fixation followed by decalcification and paraffin-embedding^8^, therefore would provide limited information for biomedical research. In this study, we sought to generate the profile of genes predominantly expressed in freshly-dissected whole cochlear tissue of *M. fascicularis*, which should include genes critical to cochlear function.

## Results

**Figure 1:**
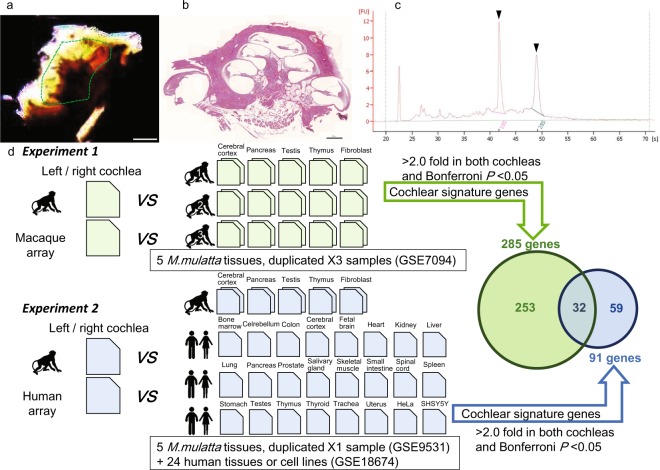
Schematic procedures to extract cochlear signature genes from *M. fascicularis*. (**a**) A dissected cochlea along with the modiolus. Tissues shown within the green dotted line were dissected out as whole cochlea and subjected to RNA extraction. Scale bar = 1 cm. (**b**) Histochemical image of a *M. fascicularis* cochlea stained with hematoxylin and eosin to show that the dissected “whole cochlea” in (**a**) corresponds to the membranous tissues of the cochlea. Scale bar = 500 μm. (**c**) Evaluation of the quality of RNA extracted from the left cochlea, as assessed with a Bioanalyzer 2100. Arrowheads indicate peaks of 18S and 28S rRNA. (**d**) Procedures of the analysis. Individual gene expression data in the left and right cochleae using the (experiment 1, top) macaque or (experiment 2, bottom) human microarray were compared with averaged expression levels of three or one macaque animals in duplicate and/or pooled human tissues or cells to extract probes that had expression levels >2-fold compared with the average of all the tissues and *P* < 0.05 (Welch’s *t*-test with Bonferroni correction). Pentagons indicate array chips.

Bilateral, whole cochlear tissues were freshly dissected from a male *M. fascicularis*, and total RNA was extracted immediately (Fig. 1a–c). We studied gene expression in the tissues and formulated a list of genes with expression levels >2-fold higher in the cochlea compared with (experiment 1) four tissues and a cell line from three independent *M. mulatta* animals^5^ on Rhesus Macaque Genome Array (macaque array) with each biological sample in duplicate; or (experiment 2) four tissues and a cell line from one *M. mulatta* animal^5^ and 22 pooled human tissues and 2 cell lines^9^ on Human Genome U133 Plus 2.0 Array (human array, Fig. 1d). Since the transcripts between *M. mulatta* and *M. fascicularis* show almost 100% identity^3^, the best platform to study gene expression in *M. fascicularis* cochlea would be macaque array. However, the platform was less frequently used (300 analyses have been registered in Gene Expression Omnibus (GEO), last visited on August 1, 2018) and predominantly for studies of viral infection or medical interventions in macaques. The number of available datasets of normal, untreated macaque tissues on the macaque array seemed limited to select genes predominantly expressed in the cochleae. The human array has been widely used (6,254 analyses in GEO, last visited on August 1, 2018) including multiple datasets of normal tissues, and was considered useful for meta-analysis to extract genes predominantly expressed in cochlear tissues. While affinities of the probes on the human array to the transcripts in the macaque tissues seemed not identical to those on macaque array^5^, the transcripts between human and *M. fascicularis* show more than 95% identity^3^, suggesting that profiles of gene expression in macaque cochleae can be analyzed on human array platforms in substitution.

Reproducibility of the datasets in the human microarray data was assessed by measuring Person’s correlation coefficient and scatter plot analyses (see Supplementary Fig. S1). Small numbers of probes showing more than 2-fold changes, high values of correlation coefficient (>0.99) between tissue replicates, and the scatter plot analyses also indicated reproducibility of the datasets in each tissue. To verify the tissue-specific gene expression in macaque cochlea, 45,902 probes detected in at least one of the macaque cochleae on human array were subjected to cluster analysis among macaque cochleae and 22 human tissues (see Supplementary Fig. S2), demonstrating that related tissues such as those in central nervous system (cortex, cerebellum, fetal brain, spinal cord) were clustered in the same group, and the macaque cochleae were closely related to the central nervous systems, suggesting that the datasets obtained in this study reflected actual profile of gene expression in the macaque cochlea.

**Figure 2:**
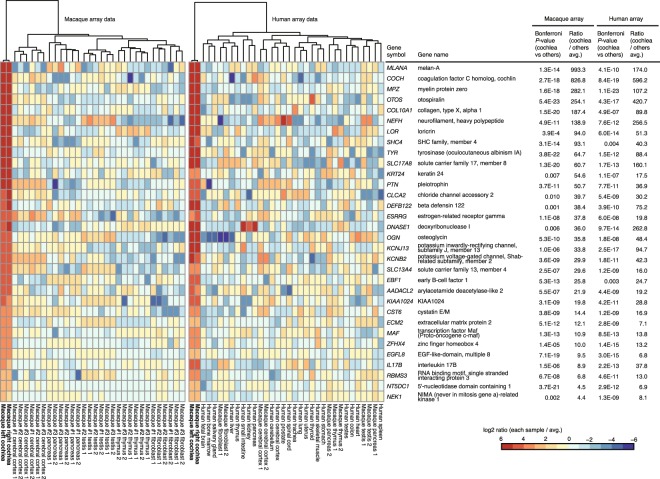
The 32 cochlear signature genes that were common to both the macaque array and the human array. Their expression levels are shown as a heat map that includes genes that clustered together in each of the macaque and human tissues. Gene symbols, gene names, Bonferroni-corrected *P*-values, and expression ratios with other tissues are shown on the right side of the map. “1” or “2” in each tissue or cell line indicates replication number.

Finally, we detected 474 probes that reflected the actual profile of 285 gene expression in experiment 1 and detected 99 probes that reflected the actual profile of 91 gene expression in experiment 2, and these genes were called cochlear signature genes (Table 1, see Supplementary Table S1). Of these genes, 32 were detected in both experiments 1 and 2, so the total number of cochlear signature genes was 344. The coincidence of the 32 genes was significant (p < 2.2 × 10^−16^, Fisher’s exact test), verifying the reproducibility of experimens 1 and 2. The “common” cochlear signature genes and the expression profile among the examined tissues are shown as a heat map in Fig. 2. Intriguingly, the cochlear signature genes included 35 genes responsible for nonsyndromic or syndromic hearing loss such as *COCH* which is responsible for autosomal dominant nonsyndroic heaing loss (DFNA9, OMIM #601369)^10^ and predominantly expressed in cochlear lateral wall, and *GJB2* which is responsible for autosomal recessive nonsyndromic hearing loss (DFNB1A, #220290)^11^, the deafness gene most frequently found world wide (Table 2). Some of other examples were; *TYR* which is associated with ocular albinism and sensorineural deafness (#103470)^12^, and *SLC17A8*, a marker gene for spiral ganglion cells in the cochlea and responsible for autosomal dominant nonsyndromic deafness (DFNA25, #605583)^13^.

**Table 1:** List of Cochlear signature genes detected on macaque or human array chip platform.

Macaque array chip			Human array chip
***MLANA***	*EBF2*	*FRMD3*	*KRTDAP*
***COCH***	*GFRA1*	***IL17B***	***COCH***
*PSMD12*	*SULF1*	*ANXA3*	*EPYC*
*SLCO2B1*	*WDR86*	*ARL9*	***OTOS***
***MPZ***	*MEPE*	*TMPRSS11E*	***NEFH***
*S100B*	*SFRP4*	*PROM1*	***DNASE1***
***OTOS***	*SLC4A11*	*PLEKHG7*	*FLG2*
*SMPX*	*LOC693624*	*RPL24*	***SLC17A8***
***COL10A1***	*ANXA4*	*DMKN*	***MLANA***
*TUFM*	***KIAA1024***	*UACA*	*SERPINB6*
*GJB6*	*TNNT1*	*EBF3*	*SLC17A6*
***NEFH***	*KIF21A*	*FGFR2*	*NDP*
*ANO5*	*OSMR*	*CPXM2*	*KCTD4*
*MLIP*	*MS4A6A*	*COLEC12*	*C17orf67*
***LOR***	*UBA6*	*ABLIM1*	***MPZ***
***SHC4***	*CRABP1*	*B3GNT5*	*NRG1*
*SLC22A2*	*BMP6*	*GSN*	***COL10A1***
*PVALB*	*SIGLEC9*	*CDK2*	*OTOR*
*ZIC2*	*MAB21L1*	*LXN*	*cDNA FLJ43186 fis*
*UGT8*	*RTFDC1*	*MEGF10*	***KCNJ13***
*SCEL*	*PPARGC1A*	*SLC26A4*	*LANCL3*
*LRP2*	*HKDC1*	*MRPS26*	***TYR***
*LOC718942*	*HTRA1*	*LASS3*	*PLEKHA4*
*SLC27A6*	*CRISP3*	*CYP26A1*	*LOC100288310*
*SLCO1A2*	*DUT*	*NIPSNAP3B*	***DEFB122***
***TYR***	*LTBP4*	*CYB5R3*	*cDNA IMAGE:1625225*
***SLC17A8***	*KRT23*	*VASH2*	***OGN***
***KRT24***	*ALDH1A3*	*S100A10*	*GJB2*
*SCIN*	*SPTLC3*	*OAS1*	*CALCA*
*SV2C*	*RDH10*	*IRX6*	*LECT1*
*GAS2*	*TM4SF18*	*IL18*	***LOR***
*SPP1*	*SMCO3*	*HPGD*	*PCP4*
*DMP1*	*CTXN3*	*SLCO1B1*	*EYA1*
*DCLK3*	*SCN7A*	*HOXD1*	***SHC4***
*Mamu_482871*	*GPR87*	*MORF4L1*	***KCNB2***
***PTN***	*ITGB8*	***RBMS3***	*IRX5*
*PheRS*	*CRTAC1*	*DACH1*	***PTN***
*SERPIND1*	*UPK1B*	*LOC696306*	***IL17B***
*CLDN8*	*MRAP2*	*MFAP3L*	***KIAA1024***
*TNFRSF11B*	*WDR18*	*FIBIN*	*POU4F2*
*DSC2*	*FIG4*	*GRHL1*	***CLCA2***
*VTCN1*	*STAC*	*TFAP2B*	*POU4F1*
*C19H19orf33*	*SCARA5*	*CPSF6*	*PLCB4*
*LOC717747*	*TNFRSF19*	*LOC720403*	*cDNA FLJ37676 fis*
*CRYAB*	*MGP*	*PIK3R1*	***EBF1***
*LGR5*	*BCAS1*	*GAL3ST1*	*C12orf69*
*OVOS*	*CLIC5*	*WDR11*	*FLG*
*IBSP*	*OLFM4*	*SCUBE2*	*KCNN2*
*CP*	*CCDC114*	*FREM1*	*LRRN1*
*PAPSS2*	*SVIP*	*C1ORF162*	*KCNQ4*
*P2RY2*	*MS4A7*	*GANC*	***AADACL2***
*KLK7*	*F13A1*	*UST*	*MAGI1*
*GJB4*	***CST6***	*ACSL1*	***KRT24***
***ESRRG***	*INSC*	*GDPD3*	*CARD18*
***CLCA2***	*CDH19*	*WNK3*	***ESRRG***
***OGN***	*RERGL*	*ERMP1*	*RAB12*
***DEFB122***	*PAX3*	*GPC6*	***SLC13A4***
*OMD*	*HEY2*	*CSRP2*	***CST6***
***SLC13A4***	*ELOVL7*	*IFIT1*	*cDNA DKFZp686P21116*
***DNASE1***	*MALL*	*MPP6*	*LOC283143*
***KCNB2***	*PPP1R1C*	*BGLAP*	*FIGN*
*OTOGL*	*PROS1*	*MPZL2*	*FAM190A*
*SSBP1*	*HHATL*	*MTMR6*	*RAD54B*
***KCNJ13***	*CALML5*	*EEF1D*	*GRIK2*
*MAPK8IP2*	*LOC693471*	*NPNT*	***RBMS3***
*DSC1*	*LDLRAD3*	*FAM162A*	***MAF***
*EYA4*	*EGR2*	*CD55*	***ZFHX4***
*FMO3*	*PTPRU*	*GTPBP8*	*LOC220077*
*PMP22*	*LOC694405*	*RORA*	*DDR2*
*TRPM1*	*EFEMP1*	*TMCC3*	*NFIB*
*TMEM213*	*IGHV4OR15-8*	***NEK1***	*AKAP12*
*ABCA10*	*PRX*	***NT5DC1***	*ITGA10*
*RDH5*	*OLR1*	*GSTM4*	*CADM1*
*PLLP*	*SERTAD4*	*DLGAP5*	*TMEM117*
*CA14*	*SPAG11*	*PXK*	*RARB*
*CA13*	***ECM2***	*CCL26*	*PLAG1*
*COL4A4*	*STX1B*	*PBX3*	*SECISBP2L*
*METTL7B*	*EIF5A2*	*COL11A2*	*LPIN1*
*ITIH2*	*SLC25A13*	*C15H9orf3*	***NEK1***
*PRH2*	*KCNE1*	*APBB2*	*ANK3*
*FILIP1*	***MAF***	*SCCPDH*	***ECM2***
*RARRES1*	*WNT16*	*EPM2A*	*SESN3*
*FOXC1*	*ZFHX3*	*TAB2*	***NT5DC1***
*SOST*	*OR51E2*	*FNTA*	*CCDC126*
***EBF1***	*ID4*	*SBF2*	***EGFL8***
*COL8A2*	*SLC5A3*		*cDNA IMAGE:3565734*
*C2ORF40*	*PFKFB3*		*ITSN1*
*ASPA*	*SOX17*		*USP32*
*CES1*	*CFB*		*DST*
*CHST9*	*WFDC5*		*PSMA2*
*COL2A1*	*ABI3BP*		*C15orf40*
***AADACL2***	*MCOLN3*		
*ABCA9*	*LOC702904*		
*MUC15*	*GPR137B*		
*CRISPLD1*	*SPTBN1*		
*IPO5*	*TM7SF2*		
*SPATA22*	*MEOX2*		
*IL20RB*	*COL9A3*		
*CTDSP2*	***EGFL8***		
*DCP2*	***ZFHX4***		

Gene symbol in bold indicates that the gene is found on both macaque and human array chip platforms.

**Table 2:** List of genes associated with nonsyndromic or syndromic hearing loss detected in this study.

Gene symbol	Disease	OMIM phenotype ID
Human and Macaque array		
*COCH*	Autosomal dominant deafness 9	601369
*MPZ*	Charcot-Marie-Tooth disease DID, type 1B, 2J	607791, 118200, 607736
*LOR*	Vohwinkel syndrome, variant form	604117
*TYR*	Albinism, ocular, with sensorineural deafness	103470
*SLC17A8*	Autosomal dominant deafness 25	605583
*MAF*	Ayme-Gripp syndrome	601088
Macaque array		
*PSMD12*	Stankiewicz-Isidor syndrome	617516
*SMPX*	Deafness, X-linked 4	300066
*GJB6*	Autosomal dominant deafness 3B, 1B	612643, 612645
*LRP2*	Donnai-Barrow syndrome	222448
*TNFRSF11B*	Paget disease of bone 5, juvenile-onset	239000
*OTOGL*	Autosomal recessive deafness 84A, 84B	613391, 614944
*EYA4*	Autosomal dominant deafness 10	601316
*PMP22*	Charcot-Marie-Tooth disease1A, 1E	118200, 118300
*COL4A4*	Autosomal recessive Alport syndrome	203780
*FOXC1*	Axenfeld-Rieger syndrome type 3	602482
*SOST*	Autosomal dominant Craniodiephyseal dysplasia, Van Buchem disease	122860, 239100
*COL2A1*	Stickler syndrome, type 1	108300
*SLC4A11*	Corneal dystrophy and perceptive deafness	217400
*MGP*	Keutel syndrome	245150
*CLIC5*	Autosomal recessive deafness 103	616042
*PAX3*	Waardenburg syndrome, type 1, 3	193500, 148820
*EGR2*	Congenital hypomyelinating neuropathy, Dejerine-Sottas disease	605253, 145900
*PRX*	Dejerine-Sottas disease	145900
*KCNE1*	Jarvell and Lange-Nielsen syndrome 2	612347
*FGFR2*	Crouzon syndrome, Pfeiffer syndrome, Apert syndrome, Antley-Bixler syndrome	123500, 101600, 101200, 207410
*SLC26A4*	Autosomal recessive deafness 4 with enlarged vestibular aqueduct, Pendred syndrome	600791, 274600
*PIK3R1*	SHORT syndrome, Immunodeficiency 36	269880, 616005
*SBF2*	Charcot-Marie-Tooth disease 4B2	604563
*COL11A2*	Autosomal dominant deafness 13, Autosomal recessive deafness 53, otospondylomegaepiphyseal dysplasia	601868, 609706, 184840
Human array		
*SERPINB6*	Autosomal recessive deafness 91	613453
*NDP*	Norrie disease	310600
*GJB2*	Audtosomal dominant deafness 3A, Autosomal recessive deafness 1A, Keratitis-ichthyosis-deafness syndrome	601544, 220290, 148210
*EYA1*	Branchiootorenal syndrome 1, Branchiootic syndrome 1	113650, 602588
*KCNQ4*	Autosomal dominant deafness 2A	600101

Gene ontology analysis of the datasets identified 434 enriched terms in the macaque array and 685 enriched terms with *P* < 0.05. As expected, groups of genes categorized to “ear development” and “ear morphogenesis” were included in the list of top 20 gene ontology categories in the macaque and human arrays, respectively (see Supplementary Table S2 and Supplementary Fig. S3).

Among the common cochlear signature genes, we attempted to compare expression levels of *COCH*, *IL17B*, and *NEK1* in the macaque cochleae with those in a human brain by quantitative RT-PCR (qRT-PCR, see Supplementary Fig. S4). Comparison of gene expression/*GAPDH* ratios indicated expression of the all three genes in the macaque cochleae was significantly higher than the human brain, partially reproducing the predominant expression of cochlear signature genes in the macaque cochlea.

## Discussion

Our study presents the profile of cochlear signature genes obtained from bilateral whole cochleae dissected from an adult male *M. fascicularis*. Based on the facts that 1) datasets detected in the macaque cochleae were suggested to reflect actual profile of gene expression by cluster analysis; 2) cochlear signature genes were enriched in genes associated with nonsyndromic or syndromic hearing loss in both microarray platforms; 3) genes categorized to ear development or ear morphogenesis were highly enriched by gene set enrichment analysis in both microarray platforms, we coclude that the method to extract cochlear signature genes using the two microarray platforms was valid.

There have been transcriptomic analyses of sensory hair cells and the progenitor cells in zebrafish lateral line^14^, regenerating chicken utricle hair cells after ototoxic drug treatment^15^, embryonic to newborn mouse inner ear sensory cells^16^ or ganglion cells^17^, or proteomic analysis of newborn mouse inner ear hair cells^18^, all of which have focused mainly on differentiation and/or regeneration of inner ear sensory hair cells or neurons. Cell type-specific analysis results in paying less attention to the surrounding non-sensory cochlear tissues, which play significant roles in normal cochlear function. Using whole cochlear tissues, we have successfully detected cochlear signature genes including *MLANA*^19^ as well as *TYR*^12^, both of which are marker genes for the melanocyte (also called as intermediate cell) in the stria vascularis. *COCH*^10^ and *GJB2*^11^, both of which are responsible for hereditary hearing loss and are expressed predominantly in the cochlear tissues other than organ of Corti were also included in the gene list, supporting the anticipation that the genes with significant roles in the cochlea show predominant expression levels in the tissues. More than 10% of the cochlear signature genes (35 out of 344) was estimated to associate with hereditary syndromic or nonsyndromic hearing loss. Since several hundreds, but not thousands of genes have been roughly predicted to associate with hereditary hearing loss^20–22^ in all the human genes (approximately 19,000–20,000)^23,24^, cochlear signature genes are presumably rich in deafness genes. Regarding the fact that novel genes associated with hearing loss have been reported every year, it raises the possibility that unreported deafness genes are included in the cochlear signature genes. One possible application of the cochlear signature genes would be to use the list to prioritize the candidate deafness genes from the results of whole exome/genome sequencing when there are no other evidence of clinical data or animal experiments associated with hearing loss.

Limitation of this study is that the gene expression data was based on bilateral cochleae from one animal, and it was not possible to conduct the statistical analysis among multiple animals to show the variance among animals. During tissue dissection, we found it extremely challenging to obtain high quality total RNA from whole cochlear tissues surrounded by thick temporal bones in macaque. During our limited opportunities to optimize how to extract RNA from several euthanized macaques, we found that only the fresh cochlear tissues dissected within 30 minutes after sacrifice and before formalin perfusion enabled recovery of total RNA with high quality (that is, RIN ≥ 7.0) for microarray analysis. The datasets presented here were obtained to minimize degeneration of RNA in the macaque cochleae and therefore valuable, even if the data came from bilateral cochleae from one individual animal. Increasing the number of macaques for the examination will enable the statistical analysis in the future and, perhaps, decrease the number of cochlear signature genes. The reason that cochlear signature genes extracted from the macaque array outnumbered those of the human array was considered to reflect the fact that a limited number of datasets was used for comparison in the case of the macaque array.

The profile of cochlear signature genes obtained from high-quality RNA, two array GeneChip platforms (including the widely used human array), and extensive comparison with five macaque tissues and 24 human tissues or cell lines constitutes a valuable resource for studies of genes that contribute to cochlear structure and function in primates, and provide insight to discover novel genes associated with hearing loss that have yet to be established in rodent models.

## Methods

### Animal care

The test animal was a 5-year-old male Malaysian *M. fascicularis* housed at the Tsukuba Primate Research Center (TPRC), National Institutes of Biomedical Innovation, Health and Nutrition (NIBIOHN), Tsukuba, Ibaraki, Japan. The animal was cared for, handled, and sacrificed according to the guidelines and regulations established by the Institutional Animal Care and Use Committee of NIBIOHN and the standard operating procedures for macaques at TPRC. The animal was housed individually in a size-appropriate cage, and the light cycle consisted of 12 h of artificial light from 7 am to 7 pm. Temperature and humidity were maintained at 25 ± 2 °C and 60 ± 10% in the animal room. The animal was fed 70 g of commercial monkey chow (Type AS; Oriental Yeast Co., Ltd., Tokyo, Japan) and 100 g of apples daily. Water was supplied *ad libitum*. All experimental procedures were approved by the Institutional Animal Care and Use Committee of NIBIOHN. Although the macaque subjected for this study had not been examined by auditory brainstem response nor by otoacoustic emissions, the animal had never shown any behaviors suspicious for hearing impairment while kept in the facility, such as ignorance to the sound. The animal was kept in the room with the environmental noise kept to below 60 dB. The animal did not have history of obesity, treatment with ototoxic drugs, nor exposure to loud sound, all of which are risk factors of age-related hearing loss^25^. Therefore, we considered that the animal had normal hearing at the time of experiment.

### Tissue collection and RNA extraction

For RNA extraction, bilateral cochleae were dissected from the test animal within 30 minutes after sacrifice by exsanguination under deep anesthesia (Fig. 1a). First, the bony labyrinths were dissected from left and right temporal bone, then connective tissues were removed and placed in ice-cold saline. RNA from whole membranous cochlear tissues was extracted using ISOGEN-II (Nippon Gene, Toyama, Japan) and purified using an RNeasy micro kit (QIAGEN, Hamburg, Germany). Quality of the RNA extracted from the cochleae was analyzed with a Bioanalyzer 2100 (Agilent Technologies, Santa Clara, CA, USA) (Fig. 1c).

### Transcription profiling

Biotinylated antisense RNA (aRNA) from 250 ng total RNA was prepared from left or right cochlea separately according to the manufacturer protocols (Affymetrix, Santa Clara, CA, USA). Then, 10 μg of aRNA was hybridized on the GeneChip Rhesus Macaque Genome Array (macaque array, Affymetrix) and the Human Genome U133 Plus 2.0 Array (human array, Affymetrix) for 16 h at 45 °C (Fig. 1d). The GeneChip microarrays were washed and stained in the Affymetrix Fluidics Station 450. The stained GeneChips were scanned using the Affymetrix Scanner 3000-7G. The images were digitized using GeneChip Operating Software (GCOS) v1.3 (Affymetrix), and the data were exported as CEL files. The microarray data were normalized using the MAS5 algorithm (Affymetrix). The intensities were converted to a logarithmic scale (base 2). To correct for bias between arrays, we then performed quantile normalization for all array data using R software (“affy” and “limma” packages). The signal reliability of each probe was determined using the MAS5 Call algorithm (Affymetrix), and each probe was assigned to one of three flags: P, present; M, marginal; A, absent (GEO #GSE111693).

In addition, the pair of gene expression data in the left and right cochleae using the macaque microarray were compared with averaged expression levels of those in four tissues and a cell line (cerebral cortex, pancreas, testis, thymus, and fibroblast, three samples with duplicated data in each tissue or cell) of *M. mulatta* using the same platform (Fig. 1c, top) (GSE7094)^5^.

The pair of gene expression data in the left and right cochleae using the human microarray were compared with averaged expression levels of those in the five tissues (one sample with duplicated data in each tissue or cell line) of *M. mulatta* (GSE9531)^5^ in addition to 24 human tissues or cell lines (bone marrow, cerebellum, colon, cortex, fetal brain, heart, kidney, liver, lung, pancreas, prostate, salivary gland, skeletal muscle, small intestine, spinal cord, spleen, stomach, testes, thymus, thyroid, trachea, uterus, HeLa, and SHSY5Y, single data in each tissue) (Fig. 1c, bottom) (GSE18674)^9^ and using the same platform. As for human tissues, total RNA of each tissue had been purchased from several providers and pooled from more than 10 individuals on average to minimize individual variations^9^. Cluser analysis was performed by Ward’s method using R.

To identify cochlear signature genes, statistical significance was assessed with Welch’s *t*-test with Bonferroni correction. Probes were extracted that had expression levels >2-fold compared with the average of all the tissues and corrected *P* < 0.05. Gene symbols were updated manually. Gene ontology analysis was conducted according to the Gene Set Enrichment Analysis software^26,27^.

### Gene expression levels measurement by qRT-PCR

Total RNA extracted from human brain (purchased from TaKaRa BIO, Shiga, Japan) or from the whole left or right cochlear tissues from the macaque was reverse transcribed by SuperScript III (ThermoFisher Scientific, Massachusetts, USA) and was subjected to qRT-PCR using PowerSybrGreen PCR Master Mix (Applied Biosystems, California, USA) and QuantStudio 3 (Applied Biosystems) according to the manufacture’s protocols. Primer sets used in this study were shown in Supplementary Table S3. The experiment was evaluated as Gene expression/endogenously expressed *GAPDH* ratio with triplicate analyses of each experiment. Statistical evaluation was done by 2-way ANOVA.

## Electronic supplementary material

Supplementary information

## Data Availability

The data described here can be found at Mutai, H. *et al*. GEO #GSE111693 (2018).
