# Supplementary material for: Gene expression dataset for whole cochlea of Macaca fascicularis
Source: Sci Rep. 2018 Oct 22;8:15554. doi: 10.1038/s41598-018-33985-9 (PMC6197234; doi:10.1038/s41598-018-33985-9)
Supplement: Supplementary file 1 — Supplementary information [file 41598_2018_33985_MOESM1_ESM.pdf]

Supplementary Information

Gene expression dataset for whole cochlea of *Macaca fascicularis*

Hideki Mutai, Fuyuki Miya, Hiroaki Shibata, Yasuhiro Yasutomi, Tatsuhiko Tsunoda, Tatsuo Matsunaga

Supplementary Information Legends

Supplementary Fig. S1. Reproducibility of the human array data. (a) Each dataset was compared with one of the datasets to show numbers of probes showing more than 2-fold changes between datasets (lower left) and to measure Person’s correlation coefficient (upper right). Number of probes and values of the correlation coefficient were color-coded as ≥ 0.95 (red), ≥ 0.90 and < 0.95 (orange), ≥ 0.85 and < 0.90 (yellow), < 0.85 (light blue) for Person’s correlation coefficient, 1-100 (red), 101-500 (orange), 501-1000 (yellow), ≥ 1001 (light blue) for # of probes showing > 2-fold change. (B-G) Scatter plot analyses between several tissue pairs. Probes with flags “P” in all the tissues (5,019 probes) were plotted.

Supplementary Fig. S2. Clustering of gene expression dynamics. Gene sets from macaque or human tissues on human array platform was analyzed based on cluster analysis by Ward’s method.

Supplementary Fig. S3. Ear development or morphogenesis-related genes in the gene set in the macaque array or the human array. Expression levels of genes categorized to “ear development”

in the macaque array (a) and “ear morphogenesis” in the human array (b) based on gene set enrichment analysis in each of the macaque and human tissues are shown as heatmaps. NES, normalized enrichment score; FDR, false discovery rate.

Supplementary Fig. S4. Gene expression of three cochlear signature genes evaluated by qRT-PCR. (a) Representative amplification plots of left macaque cochlea (*mfCOCH*, *mfIL17B*, *mfNEK1*, and *mfGAPDH*) and human brain (*hsIL17B*, *hsNEK1*). Ct value was set to be 0.1 (blue dotted line). (b) Expression levels of *COCH*, *IL17B*, and *NEK1* in a human brain and left and right whole cochlear tissues from a macaque were evaluated as ratios of gene expression /GAPDH from triplicate analyses in each sample. Data was shown as mean ± SD. *, Statistical difference ($p < 0.0001$, 2-way ANOVA).

Supplementary Table S1. List of cochlear signature genes detected on macaque or human array chip platform.

Supplementary Table S2. Top 20 gene ontology categories in the macaque or human array.

Supplementary Table S3. Primers for quantitative RT-PCR.

A

	Macaque left cochlea	Macaque right cochlea	Macaque cerebral cortex 1	Macaque cerebral cortex 2	Macaque fibroblast 1	Macaque fibroblast 2	Macaque pancreas 1	Macaque pancreas 2	Macaque testis 1	Macaque testis 2	Macaque thymus 1	Macaque thymus 2	Human bone marrow	Human cerebellum	Human colon	Human cerebral cortex	Human fetal brain	Human heart	Human kidney	Human liver	Human lung	Human pancreas	Human prostate	Human salivary gland	Human skeletal muscle	Human small intestine	Human spinal cord	Human spleen	Human stomach	Human testes	Human thymus	Human thyroid	Human trachea	Human uterus
Macaque left cochlea	1.00	0.99	0.79	0.78	0.77	0.76	0.73	0.73	0.79	0.79	0.71	0.71	0.53	0.64	0.68	0.64	0.62	0.66	0.61	0.58	0.70	0.65	0.68	0.57	0.60	0.63	0.73	0.69	0.71	0.61	0.63	0.69	0.59	0.63
Macaque right cochlea	10	1.00	0.79	0.78	0.77	0.76	0.73	0.73	0.79	0.79	0.71	0.71	0.52	0.65	0.68	0.64	0.63	0.66	0.61	0.58	0.69	0.65	0.68	0.57	0.60	0.63	0.73	0.68	0.71	0.61	0.63	0.69	0.59	0.63
Macaque cerebral cortex 1	1476	1445	1.00	0.98	0.68	0.69	0.69	0.69	0.76	0.76	0.70	0.70	0.51	0.74	0.58	0.74	0.71	0.60	0.56	0.50	0.57	0.55	0.59	0.52	0.56	0.55	0.69	0.57	0.57	0.57	0.57	0.59	0.53	0.54
Macaque cerebral cortex 2	1484	1471	40	1.00	0.68	0.69	0.69	0.69	0.76	0.76	0.70	0.70	0.51	0.73	0.58	0.74	0.71	0.60	0.56	0.50	0.57	0.54	0.58	0.52	0.56	0.54	0.68	0.57	0.57	0.57	0.56	0.59	0.53	0.54
Macaque fibroblast 1	1519	1543	1838	1830	1.00	0.99	0.76	0.76	0.76	0.77	0.74	0.74	0.63	0.58	0.75	0.57	0.58	0.66	0.65	0.68	0.71	0.71	0.71	0.66	0.61	0.71	0.65	0.72	0.73	0.66	0.68	0.69	0.68	0.68
Macaque fibroblast 2	1586	1591	1815	1798	28	1.00	0.76	0.76	0.77	0.77	0.74	0.74	0.64	0.58	0.74	0.57	0.58	0.65	0.65	0.68	0.70	0.70	0.70	0.66	0.61	0.71	0.65	0.71	0.72	0.66	0.68	0.68	0.68	0.68
Macaque pancreas 1	1805	1795	1938	1956	1540	1528	1.00	0.96	0.76	0.76	0.72	0.72	0.60	0.58	0.68	0.56	0.57	0.62	0.64	0.64	0.64	0.73	0.68	0.67	0.58	0.65	0.62	0.65	0.67	0.58	0.63	0.68	0.65	0.62
Macaque pancreas 2	1774	1781	1940	1953	1542	1530	184	1.00	0.76	0.76	0.72	0.72	0.59	0.58	0.68	0.55	0.56	0.62	0.64	0.64	0.64	0.73	0.67	0.67	0.58	0.65	0.61	0.65	0.67	0.58	0.62	0.67	0.65	0.61
Macaque testis 1	1419	1417	1788	1769	1615	1619	1683	1678	1.00	0.99	0.84	0.84	0.55	0.58	0.61	0.52	0.60	0.55	0.53	0.48	0.62	0.57	0.63	0.52	0.51	0.57	0.61	0.64	0.62	0.63	0.65	0.62	0.56	0.60
Macaque testis 2	1432	1419	1785	1776	1583	1611	1721	1707	35	1.00	0.84	0.84	0.56	0.59	0.61	0.52	0.60	0.56	0.53	0.48	0.62	0.58	0.63	0.52	0.52	0.58	0.61	0.64	0.62	0.63	0.65	0.62	0.56	0.60
Macaque thymus 1	1711	1739	1986	1937	1581	1615	1869	1870	972	940	1.00	0.99	0.64	0.56	0.62	0.49	0.57	0.51	0.51	0.50	0.60	0.57	0.60	0.52	0.52	0.58	0.56	0.67	0.60	0.60	0.76	0.59	0.54	0.54
Macaque thymus 2	1716	1735	1962	1936	1556	1567	1838	1835	984	955	35	1.00	0.64	0.56	0.61	0.49	0.57	0.52	0.50	0.50	0.59	0.57	0.59	0.52	0.52	0.57	0.55	0.67	0.59	0.61	0.76	0.59	0.54	0.54
Human bone marrow	2790	2774	2724	2732	2580	2559	2682	2648	2848	2844	2686	2683	1.00	0.72	0.81	0.71	0.72	0.68	0.78	0.72	0.77	0.76	0.76	0.80	0.67	0.83	0.70	0.82	0.75	0.73	0.85	0.74	0.84	0.80
Human cerebellum	2507	2496	2106	2135	2560	2573	2667	2633	2788	2782	2777	2765	1926	1.00	0.77	0.93	0.90	0.74	0.80	0.68	0.75	0.72	0.78	0.75	0.73	0.77	0.86	0.74	0.74	0.73	0.76	0.78	0.77	0.80
Human colon	2303	2286	2389	2399	2057	2077	2229	2232	2590	2547	2517	2546	1548	1592	1.00	0.77	0.75	0.84	0.87	0.85	0.91	0.90	0.92	0.86	0.78	0.94	0.85	0.90	0.95	0.77	0.85	0.90	0.89	0.89
Human cerebral cortex	2618	2616	2163	2203	2727	2736	2840	2803	2944	2937	2936	2954	2098	550	1835	1.00	0.89	0.75	0.82	0.70	0.75	0.72	0.77	0.78	0.73	0.79	0.87	0.72	0.73	0.72	0.73	0.77	0.79	0.80
Human fetal brain	2656	2652	2353	2359	2682	2675	2732	2728	2753	2736	2800	2760	1895	840	1779	1108	1.00	0.68	0.76	0.63	0.75	0.70	0.76	0.73	0.68	0.76	0.82	0.73	0.71	0.72	0.78	0.76	0.77	0.81
Human heart	2452	2446	2319	2350	2356	2410	2487	2484	2747	2744	2768	2774	2215	1849	1289	1927	2210	1.00	0.82	0.77	0.80	0.78	0.80	0.77	0.88	0.80	0.80	0.77	0.83	0.70	0.71	0.80	0.76	0.78
Human kidney	2649	2671	2624	2656	2552	2522	2562	2561	2931	2887	2865	2862	1689	1516	1115	1419	1774	1459	1.00	0.85	0.82	0.84	0.84	0.88	0.76	0.89	0.81	0.79	0.83	0.73	0.77	0.86	0.88	0.86
Human liver	2605	2618	2609	2648	2440	2413	2492	2474	2868	2851	2768	2786	1986	2110	1057	2109	2335	1524	1189	1.00	0.77	0.86	0.80	0.80	0.75	0.84	0.74	0.79	0.82	0.70	0.73	0.80	0.79	0.75
Human lung	2248	2269	2446	2469	2220	2258	2413	2410	2569	2559	2593	2617	1596	1643	624	1960	1719	1535	1470	1607	1.00	0.84	0.93	0.81	0.73	0.89	0.87	0.92	0.93	0.74	0.86	0.92	0.87	0.89
Human pancreas	2331	2361	2479	2496	2111	2125	2048	2066	2565	2566	2580	2581	1981	2013	773	2180	2156	1534	1432	1126	1225	1.00	0.87	0.85	0.75	0.87	0.79	0.85	0.89	0.75	0.79	0.87	0.84	0.81
Human prostate	2178	2195	2360	2371	2110	2145	2175	2200	2481	2468	2478	2511	1809	1644	577	1973	1799	1539	1431	1459	503	944	1.00	0.85	0.75	0.90	0.87	0.88	0.93	0.77	0.85	0.93	0.89	0.88
Human salivary gland	2681	2716	2717	2771	2471	2466	2447	2454	2885	2831	2792	2806	1667	1883	1303	1770	2004	1891	971	1555	1755	1316	1490	1.00	0.75	0.89	0.76	0.79	0.81	0.74	0.79	0.84	0.93	0.87
Human skeletal muscle	2561	2574	2452	2464	2426	2459	2565	2547	2782	2804	2752	2747	2268	1945	1630	1985	2231	898	1738	1584	1928	1758	1870	1871	1.00	0.77	0.73	0.72	0.75	0.70	0.71	0.75	0.73	0.75
Human small intestine	2506	2501	2621	2621	2257	2275	2447	2436	2714	2694	2690	2662	1374	1682	453	1709	1734	1712	916	1298	941	1146	886	987	1807	1.00	0.82	0.87	0.90	0.77	0.85	0.88	0.92	0.91
Human spinal cord	2237	2267	2155	2195	2429	2443	2468	2501	2655	2637	2720	2720	2027	1168	1197	1294	1511	1476	1613	1782	906	1544	985	1958	1835	1481	1.00	0.83	0.85	0.75	0.79	0.87	0.79	0.82
Human spleen	2145	2181	2380	2389	2053	2093	2252	2320	2480	2454	2343	2363	1565	1830	664	2151	1943	1646	1700	1560	523	1082	761	1777	1954	1130	1235	1.00	0.91	0.75	0.89	0.87	0.82	0.84
Human stomach	2095	2100	2339	2349	2046	2119	2192	2236	2509	2493	2488	2502	1874	1873	291	2114	2008	1371	1572	1338	480	809	502	1721	1821	897	1108	564	1.00	0.75	0.83	0.91	0.84	0.85
Human testes	2532	2545	2565	2577	2411	2406	2648	2652	2638	2642	2622	2618	2103	1989	1779	2116	2120	2131	1977	2044	1835	1924	1793	2014	2040	1789	1893	1842	1908	1.00	0.78	0.76	0.76	0.75
Human thymus	2336	2335	2473	2470	2215	2227	2384	2406	2461	2449	2076	2071	1459	1807	1173	2194	1836	2082	1842	1961	1031	1598	1124	1850	2082	1267	1562	834	1238	1777	1.00	0.85	0.83	0.83
Human thyroid	2099	2105	2318	2359	2164	2213	2229	2223	2452	2422	2534	2541	1973	1636	812	1984	1870	1536	1418	1540	561	1009	476	1518	1827	1113	988	871	673	1866	1179	1.00	0.86	0.87
Human trachea	2642	2666	2746	2767	2442	2428	2527	2549	2809	2815	2799	2789	1308	1728	1139	1712	1721	1990	1017	1660	1264	1447	1229	633	2073	735	1786	1532	1480	1915	1594	1413	1.00	0.93
Human uterus	2540	2574	2642	2618	2303	2337	2523	2587	2675	2644	2742	2705	1383	1418	1046	1584	1348	1853	1125	1808	979	1526	1069	1130	1948	847	1532	1330	1374	1909	1420	1248	733	1.00

#probes changed >2 hold: 1 - 100; 101 - 500; 501 - 1000; > 1000

Peason's correlation coefficient: 0.95 - 1.00; 0.90 - 0.94; 0.85 - 0.89; < 0.85

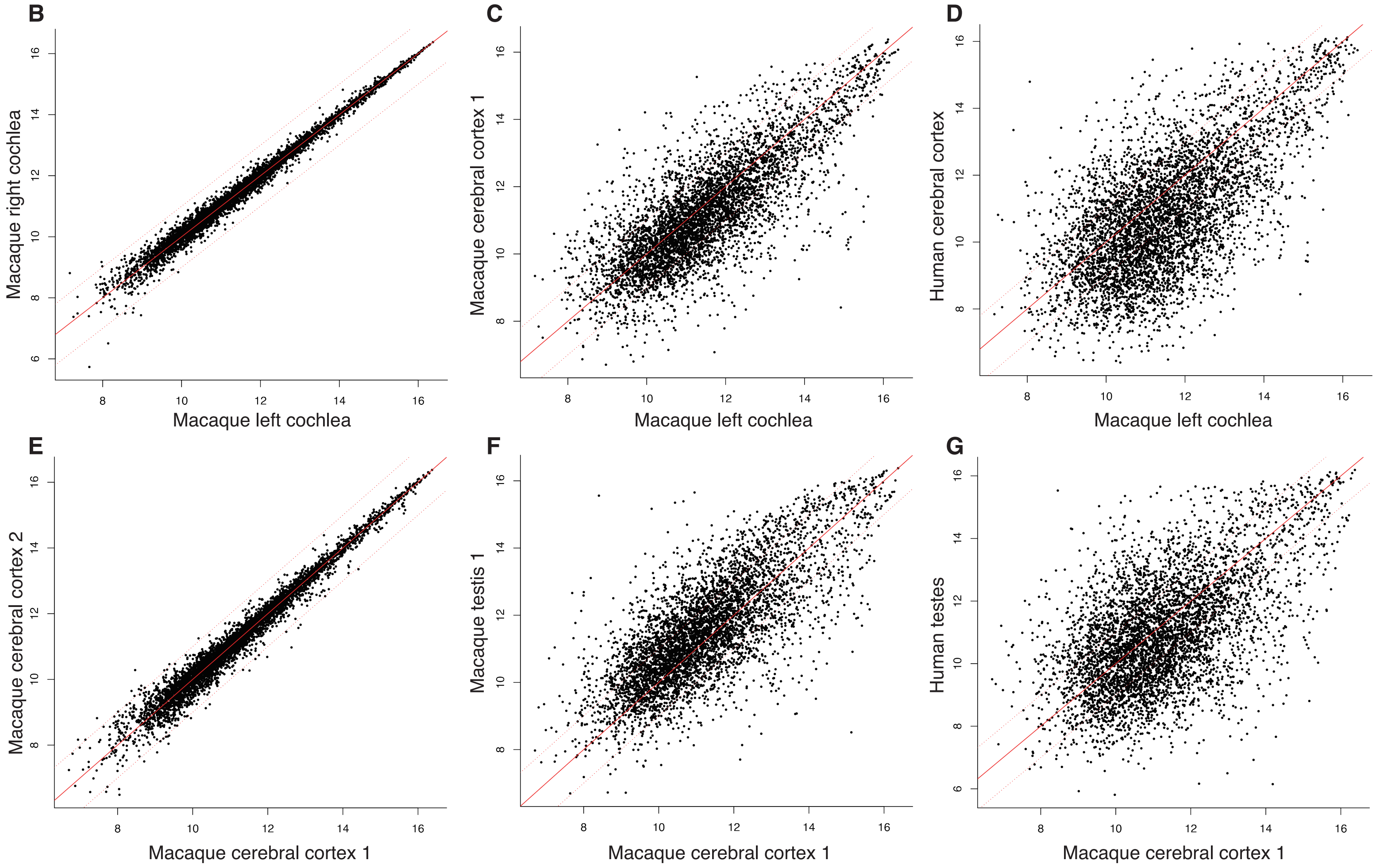

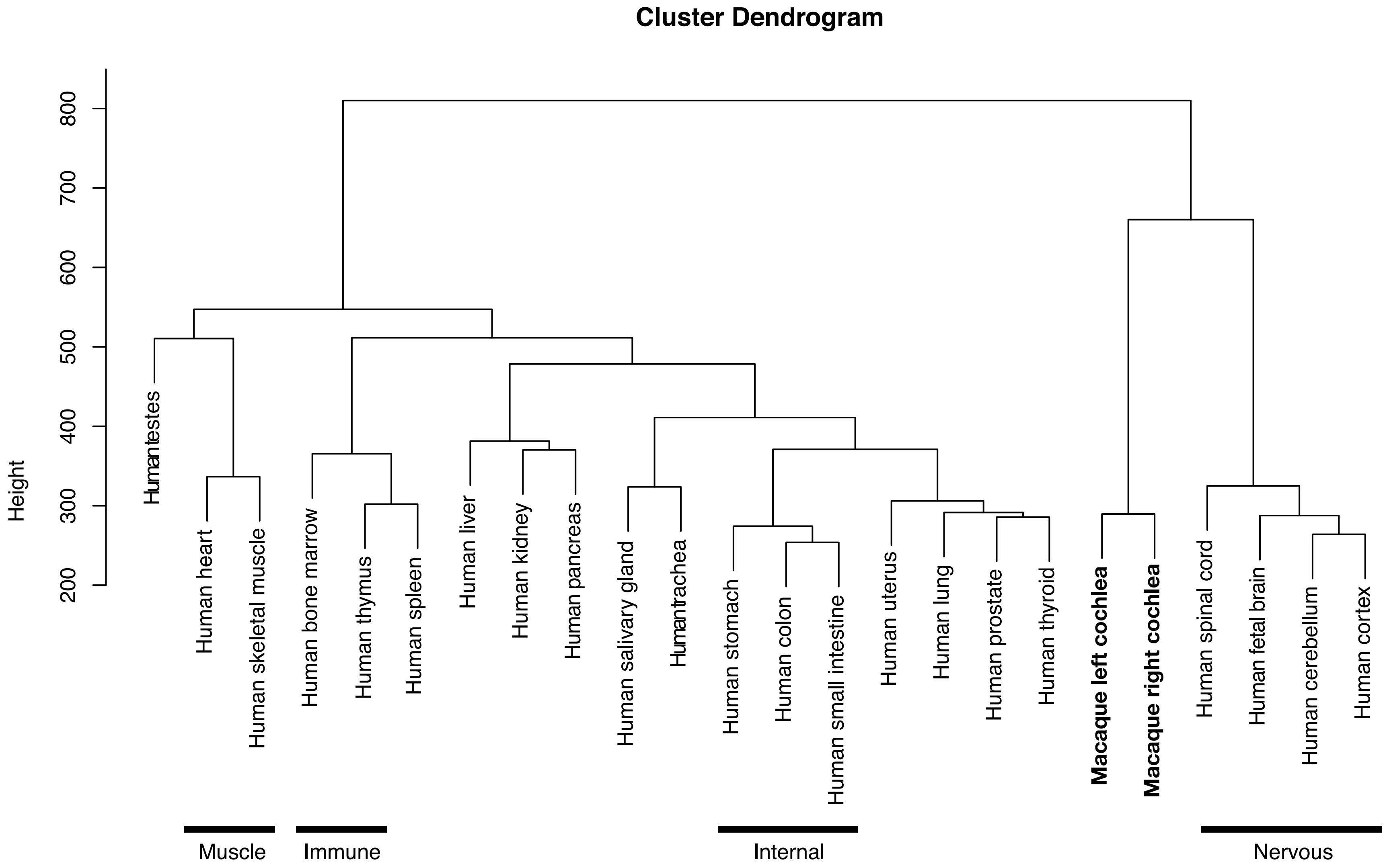

Cluster Dendrogram
Height
800
700
600
500
400
300
200
Human testes
Human heart
Human skeletal muscle
Human bone marrow
Human thymus
Human spleen
Human liver
Human kidney
Human pancreas
Human salivary gland
Human trachea
Human stomach
Human colon
Human small intestine
Human uterus
Human lung
Human prostate
Human thyroid
Macaque left cochlea
Macaque right cochlea
Human spinal cord
Human fetal brain
Human cerebellum
Human cortex
Muscle
Immune
Internal
Nervous

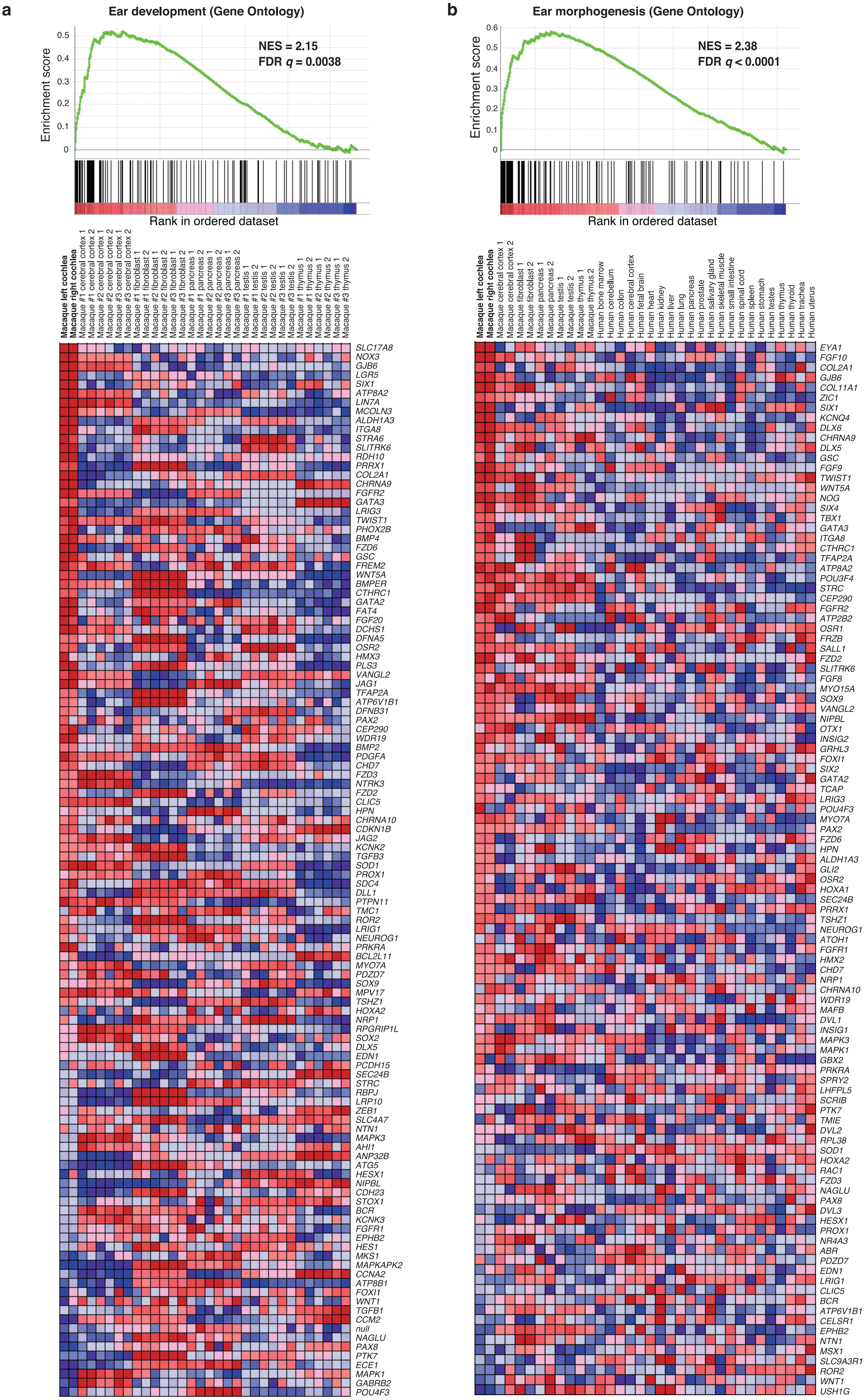
a
Ear development (Gene Ontology)
Enrichment score
NES = 2.15
FDR q = 0.0038
Rank in ordered dataset
Macaque left cochlea
Macaque right cochlea
Macaque #1 cerebral cortex 1
Macaque #1 cerebral cortex 2
Macaque #2 cerebral cortex 1
Macaque #2 cerebral cortex 2
Macaque #3 cerebral cortex 1
Macaque #3 cerebral cortex 2
Macaque #1 fibroblast 1
Macaque #1 fibroblast 2
Macaque #2 fibroblast 1
Macaque #2 fibroblast 2
Macaque #3 fibroblast 1
Macaque #3 fibroblast 2
Macaque #1 pancreas 1
Macaque #1 pancreas 2
Macaque #2 pancreas 1
Macaque #2 pancreas 2
Macaque #3 pancreas 1
Macaque #3 pancreas 2
Macaque #1 testis 1
Macaque #1 testis 2
Macaque #2 testis 1
Macaque #2 testis 2
Macaque #3 testis 1
Macaque #3 testis 2
Macaque #1 thymus 1
Macaque #1 thymus 2
Macaque #2 thymus 1
Macaque #2 thymus 2
Macaque #3 thymus 1
Macaque #3 thymus 2
b
Ear morphogenesis (Gene Ontology)
Enrichment score
NES = 2.38
FDR q < 0.0001
Rank in ordered dataset
Macaque left cochlea
Macaque right cochlea
Macaque cerebral cortex 1
Macaque cerebral cortex 2
Macaque fibroblast 1
Macaque fibroblast 2
Macaque pancreas 1
Macaque pancreas 2
Macaque testis 1
Macaque testis 2
Macaque thymus 1
Macaque thymus 2
Human bone marrow
Human cerebellum
Human colon
Human cerebral cortex
Human fetal brain
Human heart
Human kidney
Human liver
Human lung
Human pancreas
Human prostate
Human salivary gland
Human skeletal muscle
Human small intestine
Human spinal cord
Human spleen
Human stomach
Human testes
Human thymus
Human thyroid
Human trachea
Human uterus

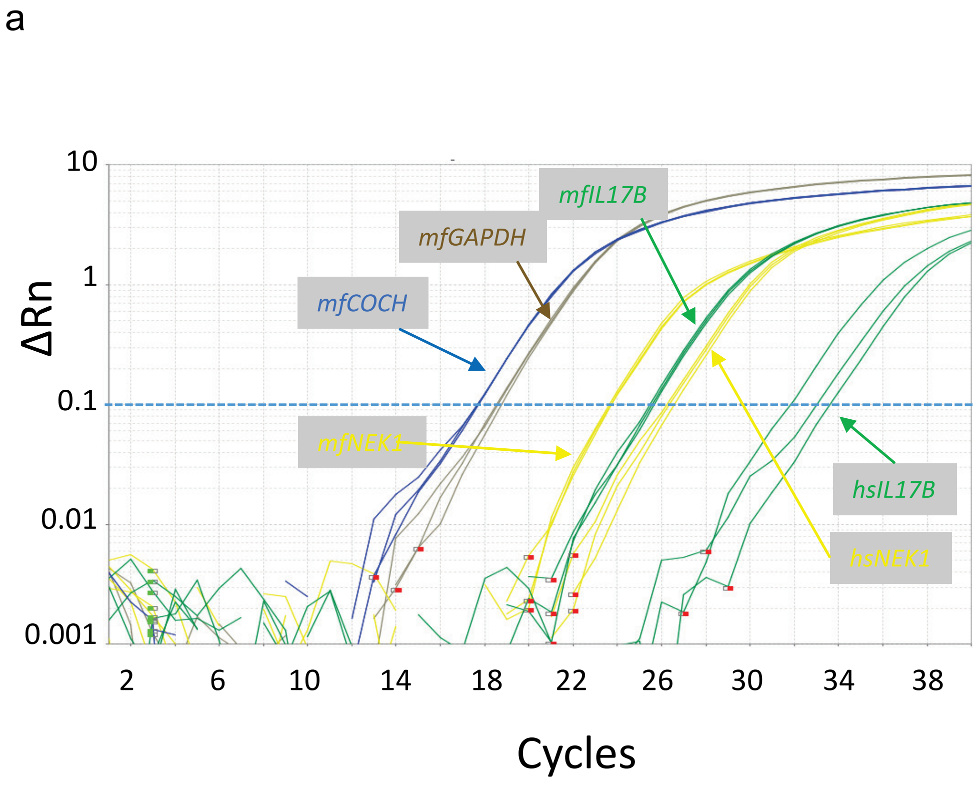

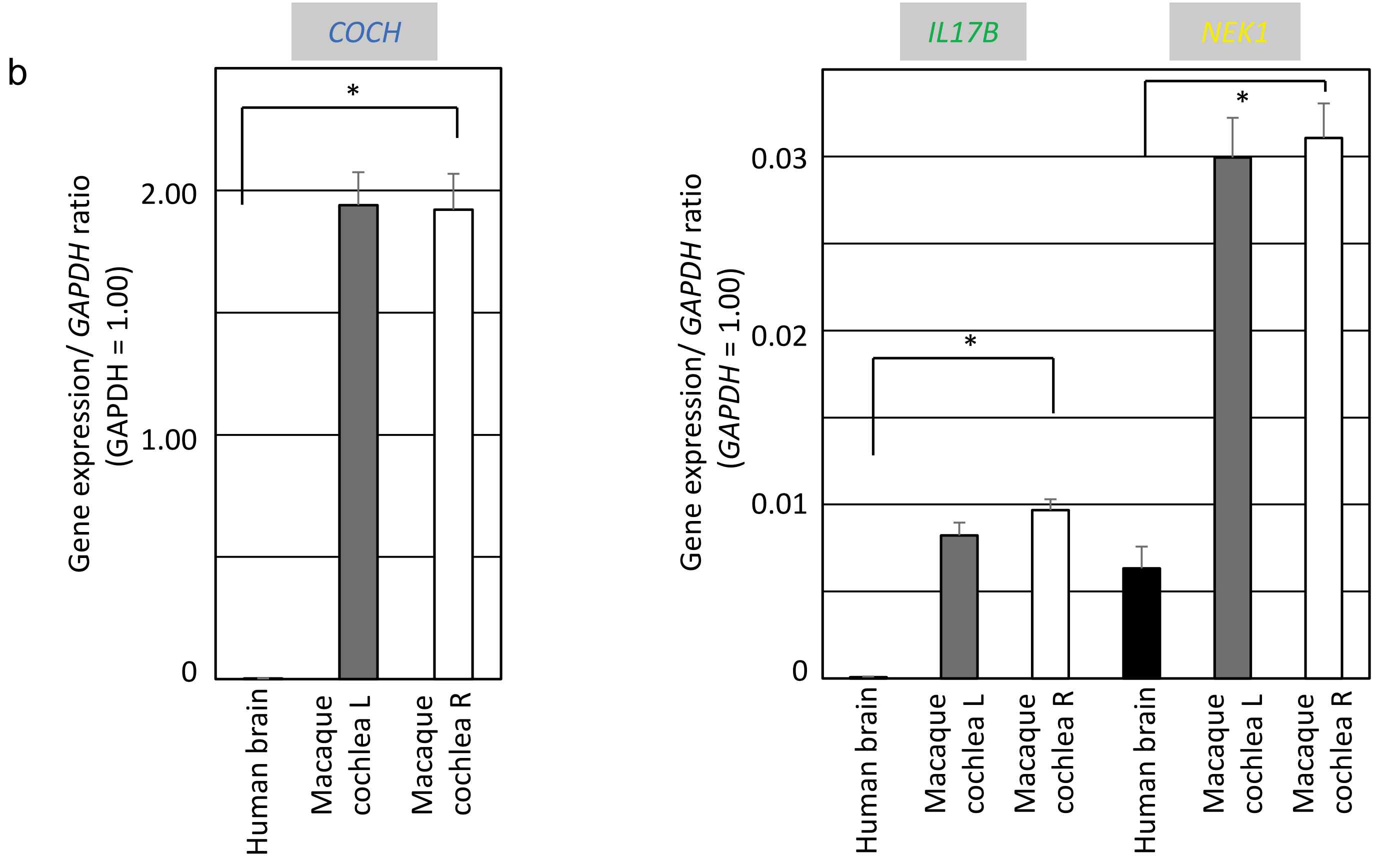

tissue	transcript	gene expression /*GAPDH* ratio
Human brain	*COCH*	(2.93 ±0.59) x10^{-3}
Macaque cochlea L	*COCH*	1.94±0.13
Macaque cochlea R	*COCH*	1.92±0.15

tissue	transcript	gene expression /*GAPDH* ratio
Human brain	*IL17B*	(7.43±3.65) x10^{-5}
Macaque cochlea L	*IL17B*	(8.23±0.73) x10^{-4}
Macaque cochlea R	*IL17B*	(9.67±0.63) x10^{-4}
Human brain	*NEK1*	(6.32±1.27) x10^{-3}
Macaque cochlea L	*NEK1*	(2.99±0.23) x10^{-2}
Macaque cochlea R	*NEK1*	(3.11±0.20) x10^{-2}

Supplementary Table S1. List of cochlear signature genes detected on macaque or human array chip platform.

Macaque array chip	Welch's t-test P value	Bonferroni Corrected P value	ratio L#	ratio R##
**MLANA**	2.4373E-19	1.2885E-14	902.496	1084.07
**COCH**	5.0372E-23	2.66291E-18	796.922	856.702
PSMD12	5.0548E-24	2.67222E-19	609.099	609.099
SLCO2B1	2.5139E-26	1.32895E-21	469.924	487.349
**MPZ**	3.0904E-23	1.63376E-18	296.929	267.179
S100B	7.7827E-15	4.11431E-10	256.912	272.824
**OTOS**	1.0309E-27	5.44968E-23	249.594	258.603
SMPX	5.727E-25	3.02759E-20	203.813	218.13
**COL10A1**	2.7775E-25	1.46831E-20	194.999	179.795
TUFM	1.3123E-15	6.9373E-11	182.818	152.665
GJB6	5.922E-14	3.13067E-09	149.414	150.898
**NEFH**	9.31E-16	4.92172E-11	134.274	143.608
ANO5	8.6962E-14	4.59726E-09	131.947	130.988
MLIP	1.3066E-17	6.90749E-13	123.796	124.708
**LOR**	7.2904E-09	0.000385408	105.99	81.9456
**SHC4**	5.7958E-19	3.06394E-14	98.8536	87.4688
SLC22A2	2.2826E-16	1.20669E-11	93.4861	80.4835
PVALB	3.0837E-13	1.63022E-08	90.6051	113.711
ZIC2	4.1673E-16	2.20304E-11	82.1819	83.7144
UGT8	8.1037E-13	4.28401E-08	78.1544	80.1974
SCEL	3.2938E-07	0.017412542	73.2255	54.059
LRP2	1.8112E-09	9.57491E-05	71.1503	86.8464
LOC718942	1.2411E-09	6.56123E-05	70.3504	53.1824
SLC27A6	1.6731E-19	8.845E-15	70.3206	69.4202
SLCO1A2	1.8116E-19	9.57686E-15	68.4015	71.6757
**TYR**	7.2651E-27	3.84068E-22	64.1216	65.1945
**SLC17A8**	2.4015E-25	1.26958E-20	62.3619	59.0456
**KRT24**	1.3318E-07	0.007040407	61.4034	47.8821
SCIN	1.0481E-16	5.54059E-12	61.3636	53.6893
SV2C	5.3667E-16	2.8371E-11	60.3559	54.2847
GAS2	1.7698E-10	9.35579E-06	57.9828	75.9034
SPP1	3.4946E-18	1.84743E-13	57.8701	61.7596
DMP1	1.6594E-12	8.77244E-08	56.7807	48.6369
DCLK3	1.1017E-17	5.82436E-13	56.5367	51.7442
Mamu_482871	5.4682E-17	2.89077E-12	52.4651	53.4537
**PTN**	6.9875E-16	3.69396E-11	51.8192	49.6794
PheRS	3.0599E-09	0.000161759	51.2014	70.1931
SERPIND1	3.8232E-19	2.02115E-14	51.083	48.7745
CLDN8	2.0414E-20	1.07917E-15	50.8784	55.0795
TNFRSF11B	8.012E-13	4.23554E-08	50.2057	50.709
DSC2	1.1873E-09	6.2769E-05	49.8622	37.5434
VTCN1	7.0108E-22	3.70628E-17	47.3202	51.2806
C19H19orf33	1.6951E-11	8.96125E-07	44.663	37.2218
LOC717747	1.5604E-09	8.24889E-05	44.0828	40.1322
CRYAB	7.8526E-18	4.15126E-13	43.9625	46.1739
LGR5	3.3729E-11	1.78311E-06	43.2088	36.7429
OVOS	2.8391E-11	1.50088E-06	41.8853	48.7287
IBSP	2.239E-17	1.18367E-12	41.8026	38.0698
CP	3.5686E-16	1.88654E-11	41.5305	45.5304
PAPSS2	6.7458E-16	3.56619E-11	40.2334	43.3263
P2RY2	3.0355E-18	1.60471E-13	38.4005	36.8923
KLK7	3.5083E-11	1.85467E-06	36.5688	29.4211
GJB4	1.3683E-12	7.23357E-08	36.4347	30.3584
**ESRRG**	2.0966E-13	1.10839E-08	35.8021	39.7198
**CLCA2**	1.8812E-07	0.009944978	35.498	43.8453
**OGN**	9.9697E-15	5.27046E-10	35.0569	36.5201
**DEFB122**	2.2423E-08	0.001185368	34.6874	42.0299
OMD	2.196E-16	1.16092E-11	33.5963	32.4215
**SLC13A4**	4.6987E-12	2.48396E-07	32.4219	26.8312
**DNASE1**	1.0955E-07	0.005791228	31.7548	40.2119
**KCNB2**	6.761E-14	3.57418E-09	31.7087	28.0813
OTOGL	5.03E-07	0.026591212	31.5056	42.3699
SSBP1	6.0902E-17	3.2196E-12	30.9344	33.1231
**KCNJ13**	1.8985E-11	1.00362E-06	30.8665	36.7966
MAPK8IP2	1.4443E-13	7.63521E-09	30.5293	35.8252
DSC1	6.9875E-14	3.69393E-09	30.4424	26.2997
EYA4	9.6452E-15	5.09895E-10	30.4181	35.0762
FMO3	1.6316E-12	8.6252E-08	29.6929	25.091
PMP22	9.2289E-20	4.87884E-15	29.1342	29.1342
TRPM1	1.3696E-15	7.24035E-11	29.0478	32.3753
TMEM213	2.7508E-12	1.45423E-07	28.0897	33.1824
ABCA10	7.0794E-15	3.74254E-10	28.0254	32.2534
RDH5	5.0639E-15	2.67703E-10	28.0112	24.7206
PLLP	1.9902E-13	1.05212E-08	27.7878	27.2526
CA14	1.9563E-18	1.03419E-13	27.4283	29.253
CA13	2.4687E-13	1.30509E-08	27.264	31.4504
COL4A4	8.4727E-15	4.47908E-10	26.8216	27.9045
METTL7B	7.3403E-15	3.88044E-10	26.7165	28.4666
ITIH2	1.8286E-12	9.66665E-08	26.459	22.9027
PRH2	1.4979E-15	7.9185E-11	25.9638	23.3666
FILIP1	3.0798E-18	1.62813E-13	25.5583	25.8952
RARRES1	6.0934E-17	3.2213E-12	25.4025	23.0808
FOXC1	9.0864E-07	0.048035489	25.3246	20.849
SOST	4.8758E-13	2.5776E-08	24.9855	21.3758
**EBF1**	9.9368E-18	5.25311E-13	24.9173	26.5933
COL8A2	4.1992E-14	2.21989E-09	24.8437	22.4826
C2ORF40	6.2253E-11	3.29101E-06	24.8211	24.6959
ASPA	4.821E-16	2.54864E-11	24.6988	24.1541
CES1	1.9742E-07	0.010436554	24.4142	32.4588
CHST9	4.1521E-09	0.000219499	24.2493	19.0387
COL2A1	4.3424E-12	2.29562E-07	23.9809	28.7849
**AADACL2**	1.0336E-11	5.46391E-07	23.8049	19.9637
ABCA9	6.4437E-09	0.000340649	23.8007	18.8109
MUC15	2.9078E-10	1.5372E-05	23.7943	29.6644
CRISPLD1	4.9148E-15	2.5982E-10	23.7232	22.8166
IPO5	6.2177E-11	3.28701E-06	23.6683	24.343
SPATA22	1.7393E-14	9.1947E-10	23.6164	20.8392
IL20RB	5.9928E-15	3.16811E-10	23.2587	20.5149
CTDSP2	8.3149E-08	0.004395687	22.8485	27.9147
DCP2	1.2413E-13	6.56215E-09	22.805	22.7753

Macaque array chip	Welch's t-test P value	Bonferroni Corrected P value	ratio L#	ratio R##
EBF2	1.4999E-20	7.92909E-16	22.5952	21.4912
GFRA1	5.6006E-14	2.96077E-09	22.106	19.276
SULF1	1.1489E-15	6.07388E-11	22.0686	24.7592
WDR86	2.9241E-11	1.54581E-06	22.0276	26.6526
MEPE	2.2846E-10	1.20778E-05	21.9186	18.3077
SFRP4	1.1769E-14	6.22178E-10	21.7389	19.1381
SLC4A11	4.2526E-09	0.000224815	21.6257	19.0969
LOC693624	1.8672E-08	0.000987098	21.2005	17.0633
ANXA4	1.8495E-14	9.77728E-10	20.9064	21.148
**KIAA1024**	5.7817E-14	3.05651E-09	20.7463	18.8946
TNNT1	8.3374E-12	4.40759E-07	20.675	23.2178
KIF21A	6.5998E-14	3.48897E-09	20.5138	18.8771
OSMR	3.4468E-18	1.82214E-13	20.3677	20.3266
MS4A6A	1.664E-13	8.79685E-09	20.3442	18.1787
UBA6	6.1543E-11	3.25347E-06	20.3244	16.9604
CRABP1	6.4302E-17	3.39931E-12	20.3114	20.2191
BMP6	3.8027E-18	2.01027E-13	20.2162	20.1067
SIGLEC9	1.7377E-17	9.18646E-13	20.0248	21.9598
MAB21L1	3.3427E-15	1.76711E-10	19.8328	22.0739
RTFDC1	3.7847E-16	2.0008E-11	19.7765	21.2867
PPARGC1A	3.0718E-13	1.62392E-08	19.6764	22.0774
HKDC1	5.1082E-07	0.0270043	19.6624	24.6436
HTRA1	6.0723E-17	3.2101E-12	19.6253	18.1534
CRISP3	2.1374E-12	1.12995E-07	19.0759	22.3092
DUT	3.9195E-10	2.07203E-05	18.9173	23.341
LTBP4	8.0775E-14	4.27017E-09	18.8018	21.4823
KRT23	3.6725E-07	0.019414718	18.597	14.9769
ALDH1A3	3.3975E-11	1.79607E-06	18.5946	21.4513
SPTLC3	4.8652E-08	0.002571962	18.5856	14.4356
RDH10	2.7419E-17	1.44951E-12	18.1089	19.6066
TM4SF18	1.2007E-12	6.34744E-08	17.7191	18.1187
SMCO3	1.1298E-17	5.97282E-13	17.3139	18.0827
CTXN3	1.7524E-13	9.26411E-09	17.1746	19.5658
SCN7A	1.1958E-10	6.32181E-06	17.1541	15.1214
GPR87	3.4903E-11	1.84513E-06	17.1148	14.4187
ITGB8	7.2911E-13	3.85444E-08	16.843	18.5503
CRTAC1	2.7443E-08	0.001450798	16.7803	13.511
UPK1B	9.4134E-18	4.97639E-13	16.7267	16.1752
MRAP2	2.7675E-13	1.46303E-08	16.1929	14.424
WDR18	3.335E-07	0.017630471	15.9357	12.2229
FIG4	2.5652E-13	1.35611E-08	15.6103	16.7342
STAC	2.6033E-16	1.37623E-11	15.5977	14.2781
SCARA5	1.107E-12	5.8522E-08	15.5624	17.4071
TNFRSF19	2.1182E-12	1.11976E-07	15.5498	18.1063
MGP	6.5187E-12	3.44611E-07	15.4969	17.9872
BCAS1	2.342E-14	1.23807E-09	15.4914	14.3005
CLIC5	1.6302E-13	8.61803E-09	15.4747	16.9608
OLFM4	7.4721E-13	3.9501E-08	15.4513	14.3516
CCDC114	1.0043E-11	5.30936E-07	15.2585	15.6632
SVIP	1.2369E-08	0.000653861	15.2438	14.4153
MS4A7	1.2554E-12	6.63647E-08	15.0628	13.7865
F13A1	2.3809E-12	1.25866E-07	14.9261	14.0519
**CST6**	7.1825E-14	3.79705E-09	14.7903	14.0071
INSC	4.6469E-14	2.45659E-09	14.5626	13.3636
CDH19	1.4408E-08	0.00076166	14.5277	11.9135
RERGL	6.7808E-11	3.58468E-06	14.2274	14.3236
PAX3	1.3525E-14	7.15002E-10	14.2036	15.1708
HEY2	5.6723E-14	2.99867E-09	14.029	13.4358
ELOVL7	1.9552E-26	1.0336E-21	13.8907	13.46
MALL	7.2361E-15	3.82536E-10	13.8799	12.4781
PPP1R1C	9.2086E-07	0.048681334	13.6957	11.3499
PROS1	5.2185E-15	2.75877E-10	13.3369	12.0262
HHATL	3.9721E-07	0.020998507	13.2415	15.7968
CALML5	1.75E-14	9.25123E-10	12.9843	11.786
LOC693471	7.6097E-12	4.02288E-07	12.8589	11.1616
LDLRAD3	3.1399E-18	1.65993E-13	12.8012	13.4349
EGR2	4.0231E-12	2.12681E-07	12.5818	13.333
PTPRU	4.8217E-11	2.54901E-06	12.5703	12.1515
LOC694405	2.7787E-13	1.46897E-08	12.4929	12.4212
EFEMP1	1.8357E-17	9.70442E-13	12.3404	12.8221
IGHV4OR15-8	4.5907E-18	2.42686E-13	12.0963	11.5803
PRX	1.0106E-17	5.34257E-13	12.0815	13.0701
OLR1	1.625E-17	8.59082E-13	12.0163	11.1446
SERTAD4	4.329E-09	0.000228854	11.9528	9.92717
SPAG11	7.1419E-15	3.77555E-10	11.8359	11.7111
**ECM2**	9.5893E-17	5.06941E-12	11.7549	12.4519
STX1B	1.5496E-11	8.19205E-07	11.5745	13.378
EIF5A2	5.8421E-13	3.08841E-08	11.485	11.0532
SLC25A13	1.6635E-07	0.008794239	11.4306	14.407
KCNE1	1.2337E-15	6.52175E-11	11.2699	10.7823
**MAF**	2.3761E-18	1.25614E-13	11.042	10.8354
WNT16	1.3813E-11	7.30231E-07	10.9593	9.54788
ZFHX3	4.1858E-16	2.21281E-11	10.9464	10.8134
OR51E2	5.503E-13	2.90915E-08	10.8048	12.2236
ID4	3.8768E-11	2.0495E-06	10.7991	10.3371
SLC5A3	3.0323E-08	0.001603006	10.7102	9.16476
PFKFB3	1.161E-11	6.13751E-07	10.6391	9.36736
SOX17	4.0312E-14	2.13112E-09	10.5082	10.7587
CFB	2.6625E-11	1.40755E-06	10.4347	9.61126
WFDC5	5.6438E-12	2.98361E-07	10.396	9.14075
ABI3BP	3.3768E-18	1.78514E-13	10.3219	9.95874
MCOLN3	1.0087E-14	5.33237E-10	10.2481	11.3081
LOC702904	7.6131E-16	4.02466E-11	10.227	10.0377
GPR137B	2.7516E-17	1.45463E-12	10.1048	10.1418
SPTBN1	4.1842E-17	2.21196E-12	10.0573	10.0219
TM7SF2	1.3244E-08	0.000700168	9.73082	11.7805
MEOX2	3.5336E-07	0.018680419	9.67586	7.79349
COL9A3	2.5724E-11	1.35988E-06	9.5038	10.9206
**EGFL8**	1.3492E-23	7.13272E-19	9.43669	9.48506
**ZFHX4**	2.6227E-10	1.38648E-05	9.36587	10.5846

Macaque array chip	Welch's t-test P value	Bonferroni Corrected P value	ratio L#	ratio R##
FRMD3	4.7411E-22	2.50638E-17	9.32742	8.94829
**IL17B**	2.7902E-11	1.47506E-06	9.2865	8.49
ANXA3	1.1112E-14	5.87442E-10	9.10758	8.68306
ARL9	1.7631E-13	9.32059E-09	8.97984	8.89015
TMPRSS11E	1.162E-15	6.14276E-11	8.9702	9.04181
PROM1	3.9262E-11	2.07557E-06	8.88582	9.87374
PLEKHG7	2.7624E-15	1.46035E-10	8.80253	8.18125
RPL24	5.9684E-10	3.15519E-05	8.73115	7.66417
DMKN	6.6011E-09	0.000348968	8.70983	7.49354
UACA	1.3429E-10	7.09932E-06	8.68296	9.38536
EBF3	1.9412E-12	1.02621E-07	8.6036	9.27082
FGFR2	6.4426E-11	3.40589E-06	8.57772	8.71102
CPXM2	5.4935E-15	2.90413E-10	8.48819	8.4756
COLEC12	8.8607E-17	4.6842E-12	8.44486	8.26118
ABLIM1	5.8507E-10	3.09298E-05	8.39802	8.79268
B3GNT5	5.9901E-09	0.000316664	8.37963	7.09414
GSN	7.6524E-20	4.04546E-15	8.1705	8.60256
CDK2	1.2711E-13	6.71955E-09	7.95499	8.15836
LXN	1.9518E-07	0.010318408	7.91249	6.5999
MEGF10	9.9861E-12	5.27918E-07	7.86958	7.01892
SLC26A4	1.0308E-13	5.44915E-09	7.68215	8.0243
MRPS26	3.9496E-12	2.08798E-07	7.64228	7.50888
LASS3	7.1217E-09	0.000376487	7.54773	6.43964
CYP26A1	5.7794E-09	0.000305526	7.51281	8.88319
NIPSNAP3B	1.3824E-18	7.30792E-14	7.39952	7.51998
CYB5R3	1.0739E-09	5.67709E-05	7.3291	7.14024
VASH2	3.1823E-09	0.00016823	7.30853	8.61359
S100A10	4.0861E-12	2.16009E-07	7.17506	6.82944
OAS1	1.9317E-13	1.02118E-08	7.16272	7.52054
IRX6	2.9924E-07	0.015819188	7.08447	8.79753
IL18	1.7925E-15	9.47625E-11	7.03344	7.38036
HPGD	1.8701E-08	0.000988621	7.03032	8.43575
SLCO1B1	9.6684E-12	5.1112E-07	7.02972	6.29671
HOXD1	6.7634E-14	3.57548E-09	6.9574	6.45682
MORF4L1	8.7357E-20	4.61815E-15	6.94073	7.18966
**RBMS3**	1.2606E-12	6.66439E-08	6.9169	6.63387
DACH1	1.6741E-13	8.85008E-09	6.86242	7.53518
LOC696306	1.3753E-17	7.27047E-13	6.82993	6.68634
MFAP3L	2.5971E-15	1.37294E-10	6.7838	6.75534
FIBIN	7.0729E-10	3.73907E-05	6.72435	6.00903
GRHL1	2.5082E-10	1.32598E-05	6.71301	6.06617
TFAP2B	1.0366E-10	5.47972E-06	6.70438	5.94008
CPSF6	1.1453E-14	6.0547E-10	6.62023	6.38033
LOC720403	1.9437E-15	1.02752E-10	6.54909	6.13526
PIK3R1	2.1795E-17	1.15219E-12	6.43572	6.06862
GAL3ST1	3.0294E-13	1.60147E-08	6.40341	6.34725
WDR11	4.3845E-10	2.31785E-05	6.374	7.22869
SCUBE2	4.1108E-09	0.000217315	6.32794	5.54822
FREM1	7.6259E-15	4.03142E-10	6.26102	6.56845
C1ORF162	1.3148E-07	0.006950756	6.2366	5.31695
SBF2	3.7265E-14	1.97003E-09	6.17043	5.74934
GANC	2.9439E-14	1.55629E-09	6.06178	5.71409
UST	3.2842E-08	0.001736176	5.95696	5.41306
ACSL1	1.3615E-19	7.19753E-15	5.94682	5.94308
GDPD3	1.9782E-12	1.04578E-07	5.93433	5.87198
WNK3	7.6223E-16	4.02952E-11	5.85825	5.61342
ERMP1	1.2707E-20	6.71762E-16	5.71924	5.69858
GPC6	2.8077E-20	1.48427E-15	5.66126	5.61083
CSRP2	1.0904E-16	5.76416E-12	5.52785	5.26127
IFIT1	4.1091E-15	2.17228E-10	5.2547	5.06089
MPP6	2.8799E-18	1.52245E-13	5.25047	5.00917
BGLAP	5.2357E-16	2.76786E-11	5.18721	5.47585
MPZL2	2.5366E-13	1.34099E-08	5.08527	5.49551
MTMR6	2.9431E-17	1.55584E-12	5.08346	4.91838
EEF1D	7.9962E-07	0.042271908	5.08149	5.70236
NPNT	3.6447E-14	1.92676E-09	4.94027	5.23162
FAM162A	3.1037E-13	1.64079E-08	4.86667	5.179
CD55	7.7598E-15	4.10222E-10	4.84429	4.5482
GTPBP8	2.0965E-17	1.10831E-12	4.73079	4.57634
RORA	7.9826E-12	4.22E-07	4.64062	4.39596
TMCC3	2.9052E-07	0.015358459	4.60092	5.32998
**NEK1**	2.9866E-08	0.001578876	4.59278	4.24164
**NT5DC1**	6.9507E-26	3.6745E-21	4.4952	4.56866
GSTM4	4.9174E-13	2.59956E-08	4.23868	4.19945
DLGAP5	9.567E-12	5.05762E-07	4.22675	4.56637
PXK	2.8643E-07	0.015142038	4.17532	4.55343
CCL26	1.1745E-18	6.20874E-14	4.12156	4.05763
PBX3	9.9736E-09	0.000527253	4.0811	3.75973
COL11A2	3.2691E-14	1.72823E-09	4.01859	4.27994
C15H9orf3	1.3129E-17	6.94078E-13	3.8045	3.95192
APBB2	4.1923E-14	2.21625E-09	3.75139	3.96042
SCCPDH	4.1506E-24	2.1942E-19	3.69659	3.67357
EPM2A	2.6953E-22	1.42487E-17	3.21353	3.18231
TAB2	2.9914E-08	0.001581408	3.07007	2.90393
FNTA	1.0074E-23	5.32584E-19	2.84727	2.84987

Human array chip	Welch's t-test P value	Bonferroni Corrected P value	ratio L#	ratio R##
KRTDAP	1.68083E-09	9.18996E-05	888.122	613.341
**COCH**	1.5397E-23	8.41834E-19	585.248	607.124
EPYC	3.92785E-07	0.021475543	568.569	798.272
**OTOS**	7.78687E-22	4.25747E-17	430.467	410.897
**NEFH**	1.39563E-16	7.63059E-12	251.964	261.095
**DNASE1**	1.7688E-18	9.67092E-14	241.817	283.691
FLG2	8.50823E-10	4.65187E-05	185.968	136.054
**SLC17A8**	3.13534E-18	1.71425E-13	160.348	159.853
**MLANA**	7.4207E-15	4.05727E-10	158.611	189.316
SERPINB6	1.49864E-11	8.19379E-07	135.256	112.25
SLC17A6	8.49412E-16	4.64416E-11	132.142	133.02
NDP	1.74345E-16	9.53232E-12	128.507	150.868
KCTD4	1.53727E-14	8.40503E-10	114.663	141.695
C17orf67	2.65451E-11	1.45136E-06	112.359	91.5315
**MPZ**	2.04311E-28	1.11707E-23	109.142	105.236
NRG1	5.13127E-14	2.80552E-09	99.6074	80.8878
**COL10A1**	8.98973E-12	4.91514E-07	97.9896	81.5836
OTOR	1.45078E-08	0.000793214	95.2925	117.257
cDNA FLJ43186 fis	6.62947E-15	3.62466E-10	94.4418	114.38
**KCNJ13**	4.54884E-22	2.48708E-17	91.2028	98.2742
LANCL3	3.35868E-19	1.83636E-14	87.9184	92.4568
**TYR**	2.69437E-17	1.47315E-12	81.7363	95.138
PLEKHA4	2.41661E-13	1.32128E-08	79.0413	67.8235
LOC100288310	8.05169E-20	4.40226E-15	78.7681	77.0698
**DEFB122**	7.14371E-15	3.90583E-10	68.9345	81.4676
cDNA IMAGE:162522	1.15665E-17	6.32398E-13	61.9358	54.6865
**OGN**	3.32773E-13	1.81944E-08	52.5929	44.2831
GJB2	3.16137E-15	1.72848E-10	51.5839	49.0735
CALCA	2.88978E-19	1.57999E-14	50.5362	46.1401
LECT1	1.72759E-07	0.009445606	49.7542	67.5979
**LOR**	1.09021E-18	5.96072E-14	49.6887	52.9544
PCP4	2.23937E-12	1.22438E-07	47.6145	45.315
EYA1	2.02698E-14	1.10825E-09	46.5943	55.143
**SHC4**	7.05844E-08	0.0038592	45.2186	35.4557
**KCNB2**	3.23773E-16	1.77023E-11	44.7496	39.7782
IRX5	5.30549E-10	2.90077E-05	40.5082	52.8447
**PTN**	1.39938E-15	7.65112E-11	37.8208	36.0706
**IL17B**	4.07615E-18	2.22864E-13	36.5613	39.026
**KIAA1024**	7.71453E-16	4.21792E-11	30.6648	26.97
POU4F2	5.22996E-12	2.85948E-07	29.5632	35.7213
**CLCA2**	9.90476E-14	5.41543E-09	27.9672	32.4475
POU4F1	1.24861E-09	6.82677E-05	27.4742	22.0864
PLCB4	6.69625E-14	3.66118E-09	24.9614	26.3412
cDNA FLJ37676 fis	1.91886E-15	1.04914E-10	23.4538	24.1428
**EBF1**	5.99926E-08	0.003280094	22.5112	26.7948
C12orf69	1.35645E-10	7.41641E-06	22.2343	26.6238
FLG	1.05637E-14	5.77572E-10	21.9563	23.1958
KCNN2	7.83853E-12	4.28572E-07	21.8257	25.9198
LRRN1	3.40611E-12	1.86229E-07	20.796	22.37
KCNQ4	1.37932E-12	7.54143E-08	20.3406	17.3875
**AADACL2**	8.12034E-14	4.4398E-09	20.2536	18.0815
MAGI1	6.6972E-14	3.66169E-09	19.6875	18.1255
**KRT24**	2.05168E-12	1.12175E-07	18.9173	16.1399
CARD18	2.94744E-10	1.61151E-05	18.2854	15.0452
**ESRRG**	1.0951E-12	5.98746E-08	18.1978	21.3551
RAB12	4.4215E-15	2.41745E-10	16.9902	17.529
**SLC13A4**	2.28459E-14	1.2491E-09	16.9471	15.0891
**CST6**	2.18993E-14	1.19735E-09	16.2056	17.6936
cDNA DKFZp686P21116	9.90448E-15	5.41527E-10	16.1695	15.5329
LOC283143	8.20985E-09	0.000448873	15.2179	18.4326
FIGN	2.02783E-11	1.10872E-06	15.1744	17.8588
FAM190A	1.28817E-13	7.04306E-09	14.5886	16.0655
RAD54B	7.16338E-14	3.91658E-09	14.1095	12.479
GRIK2	2.43561E-11	1.33167E-06	14.0994	16.733
**RBMS3**	8.40303E-16	4.59435E-11	13.6286	12.4059
**MAF**	1.55059E-17	8.47788E-13	13.3892	14.1447
**ZFHX4**	2.48477E-20	1.35855E-15	13.0827	13.3511
LOC220077	2.16765E-17	1.18516E-12	12.4089	12.0712
DDR2	2.1042E-15	1.15047E-10	10.7285	11.2414
NFIB	1.38257E-14	7.55921E-10	10.6059	11.326
AKAP12	6.76084E-07	0.036964914	10.4478	12.6929
ITGA10	4.91479E-20	2.68716E-15	10.3118	10.4868
CADM1	7.25089E-13	3.96442E-08	9.254	9.97276
TMEM117	4.66784E-13	2.55214E-08	8.86899	9.17489
RARB	7.72344E-18	4.22279E-13	8.3472	7.84398
PLAG1	4.37439E-15	2.3917E-10	8.22848	8.97756
SECISBP2L	5.71478E-19	3.12455E-14	8.08508	8.00155
LPIN1	1.93191E-19	1.05627E-14	8.06805	7.91743
**NEK1**	2.36263E-14	1.29177E-09	7.73133	8.42121
ANK3	5.12272E-13	2.80085E-08	7.08822	6.5178
**ECM2**	5.09419E-14	2.78525E-09	7.07873	7.03779
SESN3	2.00977E-17	1.09884E-12	7.04438	6.65149
**NT5DC1**	5.28447E-17	2.88929E-12	6.88575	6.85648
CCDC126	3.14782E-12	1.72107E-07	6.77333	6.13155
**EGFL8**	5.48777E-20	3.00044E-15	6.70883	6.9014
cDNA IMAGE:356573	9.2714E-16	5.06914E-11	6.60608	6.59358
ITSN1	8.11169E-18	4.43507E-13	6.15232	6.03912
USP32	6.73529E-18	3.68252E-13	5.91377	5.71854
DST	4.61144E-14	2.5213E-09	5.74578	5.94433
PSMA2	2.54026E-24	1.38889E-19	5.72438	5.912
C15orf40	2.38173E-15	1.30221E-10	3.14364	3.0141

Genes found on both macaque and human array chip platforms are shown in bold letters.
#Ratio of expession levels in left cochlea versus tissue average. ##ratio of expession levels in right cochlea versus tissue average.

Supplementary Table S2. Top 20 gene ontology categories in the macaque or human array.

GO NAME	SIZE	NES#	FDR## q-val
Macaque array			
PROTEINACEOUS EXTRACELLULAR MATRIX	233	2.2	7.5E-03
BASEMENT MEMBRANE	64	2.2	5.4E-03
EXTRACELLULAR MATRIX	270	2.2	4.3E-03
EAR DEVELOPMENT	116	2.2	3.8E-03
BONE MINERALIZATION	23	2.1	4.9E-03
CALCIUM DEPENDENT PHOSPHOLIPID BINDING	33	2.1	5.7E-03
SENSORY PERCEPTION OF MECHANICAL STIMULUS	94	2.1	4.9E-03
BIOMINERAL TISSUE DEVELOPMENT	44	2.1	7.0E-03
EPIDERMIS DEVELOPMENT	130	2.0	1.2E-02
EXTRACELLULAR MATRIX COMPONENT	87	2.0	1.1E-02
NEURON PROJECTION MORPHOGENESIS	242	2.0	1.1E-02
CALCIUM ION BINDING	384	2.0	1.3E-02
SYNAPSE ORGANIZATION	83	2.0	1.3E-02
CONNECTIVE TISSUE DEVELOPMENT	123	2.0	1.6E-02
UROGENITAL SYSTEM DEVELOPMENT	182	2.0	1.9E-02
EXTRACELLULAR MATRIX STRUCTURAL CONSTITUENT	48	2.0	1.9E-02
EMBRYONIC CAMERA TYPE EYE DEVELOPMENT	24	2.0	1.8E-02
CELL MORPHOGENESIS INVOLVED IN NEURON DIFFERENTIATION	226	2.0	1.7E-02
CARTILAGE DEVELOPMENT	98	2.0	1.6E-02
CELL PART MORPHOGENESIS	356	2.0	1.7E-02
Human array			
TRANSMISSION OF NERVE IMPULSE	50	2.5	0
SYNAPSE ORGANIZATION	138	2.4	0
REGULATION OF SYNAPSE ORGANIZATION	103	2.4	0
VOLTAGE GATED ION CHANNEL ACTIVITY	177	2.4	0
ACTION POTENTIAL	89	2.4	0
EAR MORPHOGENESIS	105	2.4	0
VOLTAGE GATED POTASSIUM CHANNEL ACTIVITY	86	2.4	0
MULTICELLULAR ORGANISMAL SIGNALING	119	2.4	0
CORNIFIED ENVELOPE	23	2.4	0
DELAYED RECTIFIER POTASSIUM CHANNEL ACTIVITY	36	2.4	0
CATION CHANNEL ACTIVITY	264	2.4	0
SENSORY ORGAN MORPHOGENESIS	226	2.3	0
GATED CHANNEL ACTIVITY	293	2.3	0
VOLTAGE GATED CATION CHANNEL ACTIVITY	128	2.3	0
CATIONvCHANNEL COMPLEX	157	2.3	0
INNER EAR MORPHOGENESIS	86	2.3	0
NEURONAL ACTION POTENTIAL	25	2.3	0
NEURON PROJECTION MORPHOGENESIS	357	2.3	0
SENSORY PERCEPTION OF MECHANICAL STIMULUS	136	2.3	0
POTASSIUM CHANNEL COMPLEX	89	2.3	0

#NES, normalized enrichment score; ##FDR, false discovery rate

Supplementary Table S3. Primers for quantitative RT-PCR

Gene symbol	**transcript ID**	**Forward primer (5'->3')**	**Reverse primer (5'->3')**
mfCOCH and *hsCOCH*	XM_005561022.2, NM_001135058.1	TCCAATACAGGAAAAGCCTTGA	ACCACTTTGGGGATCCCTTT
mfIL17B	XM_015451486.1	TACAGCATCAACCACGACCC	TTCACACAGCCCAGACACAG
hsIL17B	NM_014443.2	GGCTACAGCATCAACCACGA	TTCACACAGCCCAGACACAG
mfNEK1	XM_015451026.1	TTATGGGGCTGCAGGTCATC	TAGCTGCCCTTTTTGCCTGT
hsNEK1	NM_001199397.1	CCGAGTTCAGTGAGGCATCT	TGTTTGTTCCACTTGGCTCTTG
mfGAPDH and *hsGAPDH*	NM_001319428.1, NM_002046.6	CACCATCTTCCAGGAGCGAG	GACTCCACGACGTACTCAGC

mf, *Macaca fascicularis*
hs, *Homo sapiens*
